# Preparation and Characterization of Polyurethanes with Cross-Linked Siloxane in the Side Chain by Sol-Gel Reactions

**DOI:** 10.3390/ma10030247

**Published:** 2017-02-28

**Authors:** Hui Zhao, Tong-Hui Hao, Guo-Hua Hu, Dean Shi, Da Huang, Tao Jiang, Qun-Chao Zhang

**Affiliations:** 1 Hubei Collaborative Innovation Center for Advanced Organic Chemical Materials, Ministry of Education Key Laboratory of Green Preparation and Application for Functional Materials, School of Materials Science & Engineering, Hubei University, Wuhan 430062, China; zhh0625@foxmail.com (H.Z.); haoth@hubu.edu.cn (T.-H.H.); deanshi2012@yahoo.com (D.S.), ganminzhao@163.com (D.H.); jiangtao@hubu.edu.cn (T.J.); 2 Laboratory of Reactions and Process Engineering, CNRS-University of Lorraine, 1 rue Grandville, BP 20451, Nancy CEDEX 54001, France; guo-hua.hu@univ-lorraine.fr

**Keywords:** cross-linked, polysiloxane, polyurethane, micromorphology

## Abstract

A series of novel polyurethanes containing cross-linked siloxane in the side chain (SPU) were successfully synthesized through a sol-gel process. The SPU was composed of 0%–20% *N*-(n-butyl)-3-aminopropyltriethoxysilane (HDI-T) modified hexamethylene diisocynate homopolymer. The effects of HDI-T content on both the structure and properties of SPU were investigated by Fourier transform infrared spectroscopy (FT-IR), X-ray photoelectron spectroscopy (XPS), X-ray diffraction (XRD), scanning electron microscopy (SEM), energy dispersive spectroscopy (EDS), differential scanning calorimetry (DSC), thermogravimetric analysis (TGA), mechanical properties tests, gel content test, water contact angle measurement and water absorption test. FT-IR, XPS and XRD results confirmed the successful incorporation of HDI-T onto polyurethanes and the formation of Si–O–Si. The surface roughness and the Si content of SPU enhanced with the increase of HDI-T content. Both crystallization and melting temperature shifted to a lower point after the incorporation of HDI-T. The hydrophobicity, tensile strength, Young’s modulus and pencil hardness overall increased with the increasing of HDI-T content, whereas the thermal stability and the elongation at break of SPU slightly decreased.

## 1. Introduction

Polyurethanes (PUs) are used in numerous applications such as coatings, adhesives, fibers, foams, rubbers, thermoplastic elastomers and composites [1,2,3,4,5,6,7]. It is the flexibility in selection of monomers from a great variety of diisocyanates, polyester and chain extenders, as well as the ability to form different types of molecular architectures that provide PUs with many excellent properties such as toughness, abrasion resistance, mechanical properties and chemical resistance [8,9,10,11,12].

However, there is still a need to improve properties of PUs with the emphasis on their hydrophobicity and mechanical properties. This can be achieved by varying the microstructures of PUs or incorporating inorganic fillers. For example, organic-inorganic nanocomposites were developed to combine the desirable properties of PUs and those of inorganic fillers [13,14,15,16] such as clay, silica and other nano-sized layered silicates. Consequently, significant improvement in performances such as mechanical properties, thermal stability and others was achieved. Moreover, various PU/polysiloxane hybrids, PU/alkoxysilane hybrids, PU/acrylic hybrids, PU/epoxy resin hybrids, were prepared to offer synergetic proprieties through different methods, such as ultraviolet polymerization, miniemulsion polymerization, seeded emulsion polymerization and interpenetrating polymer networks [17,18,19,20].

Among the above methods, modification of PUs with polysiloxane is an important and effective way to prepare high performance materials [21]. The unique properties of polysiloxane, such as low surface energy, good thermal stability, and excellent flexibility, are mainly attributed to its intrinsic structure contained inorganic Si–O bonds. The combination of PU and polysiloxane could offer a better heat resistance and low temperature flexibility than PU alone and better mechanical properties and abrasion characteristics than polysiloxane alone [22,23,24]. Therefore, the possibility of combining the advantages of polysiloxane and PU has triggered many investigations for a long time.

**Figure 1:**
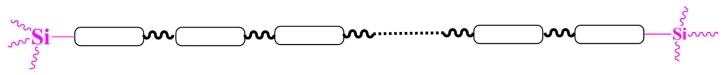
Modification of PU backbone with siloxane.

Most methods for improving water resistance, surface hydrophilicity and mechanical strength of PU are to introduce siloxane at both ends of the PU chain [25,26,27,28,29,30,31,32,33,34,35], as shown in Figure 1. The entrained Si–O–R will ensure the cross-linking of the modified PU, which may improve its water resistance, surface hydrophilicity and mechanical properties. However, the siloxane can only be introduced at the terminal groups of the backbone. Therefore, the amount of cross-linked units is low and consequently the improvement in the properties of PU may not be significant enough.

**Figure 2:**
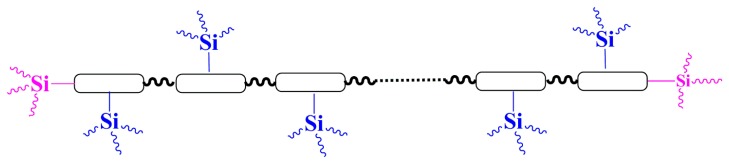
PU modified with siloxane in both backbone and side chains.

This work aims to attach siloxane onto both the backbone and side chains of PU, as shown in Figure 2. In this way, the amount of siloxane in PU would increase greatly, which may further significantly improve the surface hydrophilicity and mechanical properties.

Firstly, hexamethylene diisocyanate homopolymer (HDI-3) was grafted with *N*-(n-butyl)-3-aminopropyltriethoxysilane (NBAPTS) to generate HDI-T. A given amount of HDI-T was then mixed with 1,6-hexamethylene diisocyanate (HDI). The mixture reacted with polytetramethylene glycol (PTMG). The resulting product was chain-extended with 1,4-butanediol (BDO) and blocked with NBAPTS. This process offers a novel self-designing approach to develop novel PU with excellent properties. The structure and properties of the resultant materials were investigated by Fourier transform infrared spectroscopy (FT-IR), X-ray photoelectron spectroscopy (XPS), X-ray diffraction (XRD), scanning electron microscope (SEM), differential scanning calorimetry (DSC), thermogravimetric analysis (TGA), and mechanical and gel content tests.

## 2. Materials and Methods

### 2.1. Materials

1,6-hexamethylene diisocyanate (HDI), hexamethylene diisocyanate homopolymer (HDI-3), and polytetramethylene glycol (PTMG; number-average molecular weight *M*_n_ of 2000 g/mol were obtained from Bayer Material Science (Pittsburgh, PA, USA). *N*-butyl-3-ammonia propyl triethoxy silane (NBATPS) was of analytical purity and obtained from Hubei Diamond Advanced Material of Chemical, Inc. (Yingcheng, China). Dibutylamine, sodium bicarbonate, tetrahydrofuran (THF) and 1,4-butanediol (BDO) were analytical reagents and obtained from Sinopharm Chemical Reagent Co., Ltd. (Shanghai, China). Both PTMG and THF were dehydrated before use.

### 2.2. Experiments

#### 2.2.1. Preparation of HDI-3 Grafted NBATPS

**Figure 3:**
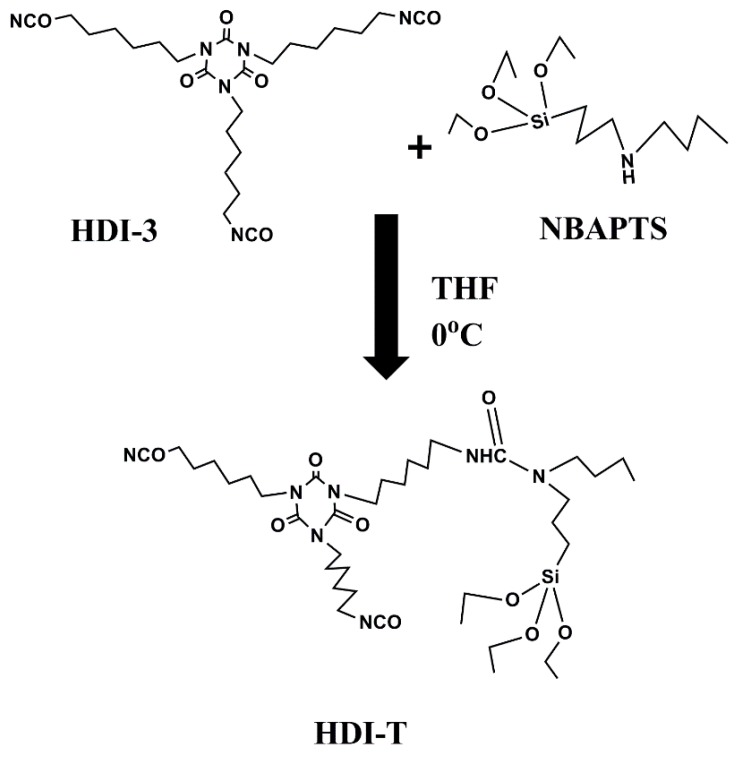
Preparation of HDI-T.

As shown in Figure 3, NBAPTS (13.85 g) and HDI-3 (27.39 g, –NCO = 23%) were added to anhydrous THF (41.24 g) in a 500 mL four-neck round-bottom flask filled with argon and equipped with a mechanical stirrer. Experiments were performed at 0 °C and the rotation speed of the mechanical stirrer was 300 rpm. The dibutylamine method was used to determine the content of the isocyanate groups in the reaction system. The reaction was stopped when the content of the isocyanate groups reached a theoretical value (–NCO = 5.09%) and the target HDI-T was obtained.

#### 2.2.2. Synthesis of Silicone/Polyurethane (SPU) Hybrids

**Figure 4:**
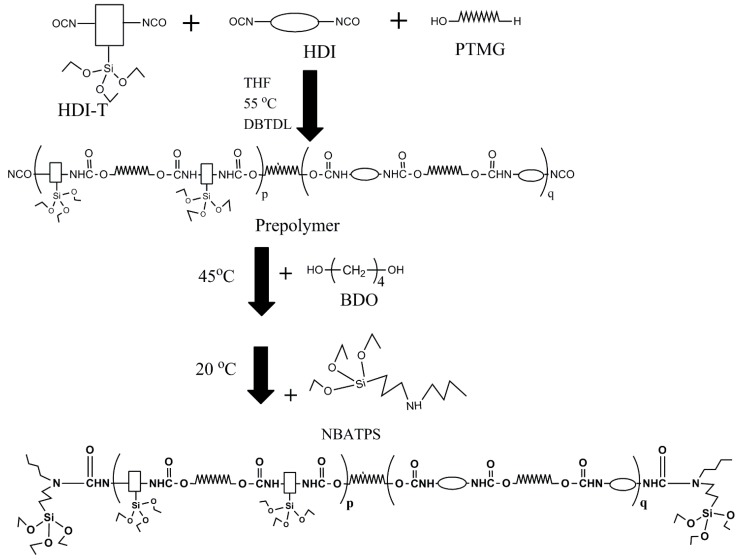
Preparation of SPU hybrids.

As shown in Figure 4, the molar ratio between isocyanate and hydroxyl groups was fixed at 1.2. THF, PTMG, HDI and HDI-T were added to a four-neck round-bottom filled with argon. The mixture was maintained at 25 °C and stirred for 30 min. Two drops of dibutyltin dilaurate (DBTDL) as a catalyst was added and the mixture was heated up to 55 °C and was maintained at that temperature until the theoretical –NCO value was reached. The resulting prepolymer was chain-extended with BDO at 45 °C. After the flask was cooled to room temperature, NBATPS was added dropwise to the flask at 20 drops per minute until the isocyanate groups were used up. Finally, a homogeneous and transparent SPU hybrid was obtained. The compositions of all samples are given in Table 1.

#### 2.2.3. Preparation of Silicone/Polyurethane (SPU) Films

**Figure 5:**
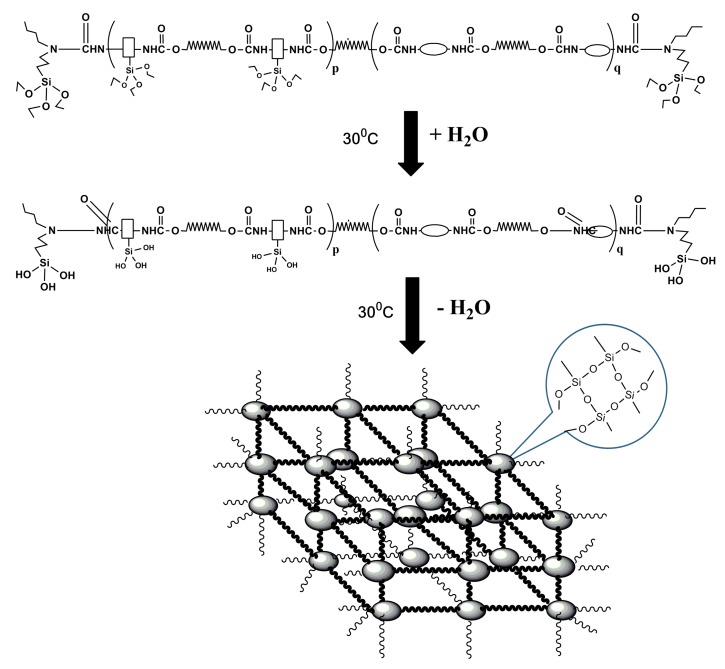
Preparation of SPU films.

All hybrids obtained in Section 2.2.2 were smeared evenly on a polytetrafluoroethylene mold at room temperature for 7 days for moisture-induced curing and a series of cross-linked SPU films were obtained, as shown in Figure 5.

### 2.3. Characterization

FT-IR spectroscopy analysis: FT-IR spectroscopy was performed on a spectrometer (Thermo Fisher Scientific, Waltham, MA, USA) using the attenuated total reflectance technique. Data were collected in the range of 4000–500 cm^−1^ at 4 cm^−1^ resolution.

XPS analysis: X-ray photoelectron spectroscopy (XPS) measurements were made on a Thermo VG ESCALAB 250 spectrometer (East Grinstead, UK) with a monochromatic Al-Kα X-ray source.

Differential Scanning Calorimetry (DSC) analysis: Between 5 and 10 mg pre-dried samples were analyzed by a DSC equipment (Q200, Newcastle, TA, USA). Samples were heated up from 30 to 100 °C at a rate of 20 °C·min^−1^ and held at 100 °C for 3 min to remove the thermal history under a dry helium purge. They were then cooled down to −50 °C at a rate of 20 °C·min^−1^. Finally, they were heated up to 100 °C again at the same rate.

Wide angle XRD analysis: Wide angle X-ray diffraction measurement was carried out with a Philips X’pert-PRO (PANalytical, Holland) using Cu-Kα radiation. The diffraction angle 2θ ranged from 5° to 60°.

Surface morphology analysis: The surface morphology of the SPU samples was analyzed by scanning electron microscopy (SEM, JSM5900LV, JEOL, Tokyo, Japan) at an accelerating voltage of 25 kV. Samples were adhered to aluminum sample holders and sputter coated with Au layer. The content of Si element on the surface of SPU films was measured by energy dispersive spectroscopy (EDS, EMAX, and EX-450 JEOL, Tokyo, Japan).

Thermogravimeter analysis (TGA): TGA was used to measure the weight loss of the SPU films under nitrogen atmosphere. Samples were heated from 100 to 700 °C at a heating rate of 10 °C·min^−1^.

Mechanical properties analysis: The tensile properties of the films were measured at 25 °C with a universal testing machine (CMT, SANS, Shenzhen Sans Material Test Instrument Co., Ltd., Shenzhen, China) at a crosshead speed of 300 mm·min^−1^. The reported values were averages of five specimens. Pencil hardness was carried out by a pencil according to ISO 15184. The SPU were coated on a glass and the final thickness of the films was about 100 µm.

Gel contents analysis: Samples (approximately 1 g) were cut from the SPU films, weighed, and then put in a Soxhlet extractor (Wuhan, China) filled with THF for 24 h. After the extraction, the samples were dried, the gel contents *W*_g_ (%), were calculated as

*W*_g_ (%) = *m*_2_/*m*_1_ × 100%. (1)

where *m*_1_ and *m*_2_ are the masses of dried SPU films before and after the extraction, respectively.

Water contact angle analysis: Water contact angles (WCA) were measured through the sessile drop method on Dataphysics OCA20 (Wuhan, China) contact angle meter. The reported WCA values were the averages of five measurements taken at five different surface locations. 

Water absorption analysis: Two grams of pre-weighed dry SPU films were immersed in de-ionized water at room temperature. After the excess water was wiped from the film surface by filter paper, the mass of the swollen film was measured immediately. The water absorption was calculated as the mass percentage of water in the swollen sample as:

Water absorption = (*m*_3_ − *m*_4_)/*m*_4_ × 100% (2)

where *m*_4_ and *m*_3_ are the masses of the dry and swollen samples, respectively.

## 3. Results and Discussion

### 3.1. Infrared Spectroscopy

**Figure 6:**
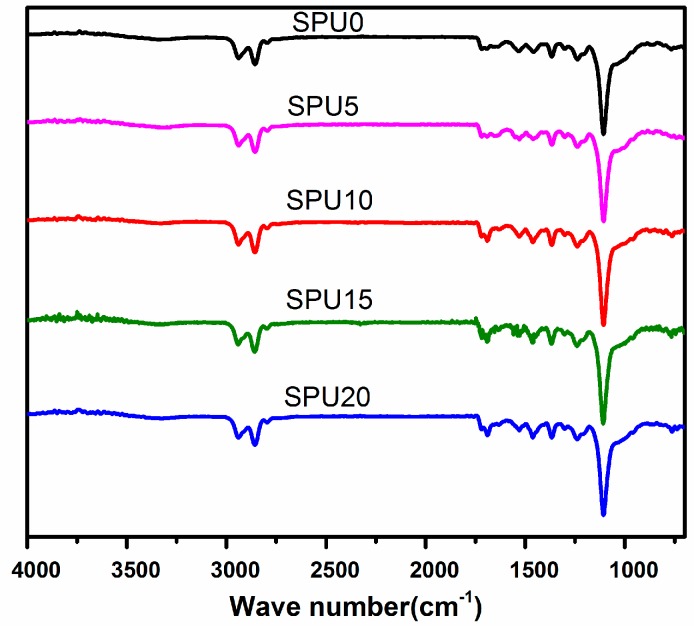
FT-IR of different SPU films.

A series of SPU hybrids and films were synthesized on the basis of the HDI-T and NBATPS. Stable hybrids and films were obtained by the addition of 0, 5, 10, 15 and 20 mol % HDI-T to SPU, respectively. The FT-IR spectra of the SPU films are shown in Figure 6. The weak absorption bands around 3310 cm^−1^ (N–H stretching) and 1530 cm^−1^ (N–H bending) and strong absorptions at 1700 cm^−1^ (free C=O stretching of urethane and carboxylic groups) and 1210–1240 cm^−1^ (stretching vibration of N–CO–O) confirm the formation of the urethane linkage. The peaks at 2938, 2857, and 2800 cm^−1^ (CH_2_ and CH_3_ stretching vibration); 1105 cm^−1^ (C–O–C stretching vibration of PTMG and Si–O–Si asymmetric stretching vibration); 1258 cm^−1^ (CH_3_– in Si–CH_3_ symmetric bending), 1072 and 1021 cm^−1^ (Si–O stretching); and 800 cm^−1^ (Si–C stretching) can clearly be observed in the spectra. The peak at 2270 cm^−1^ (N=C=O stretching) has disappeared. This indicates that siloxane groups were successfully introduced into the SPU hybrid films.

### 3.2. XPS Analysis of SPU Films

**Figure 7:**
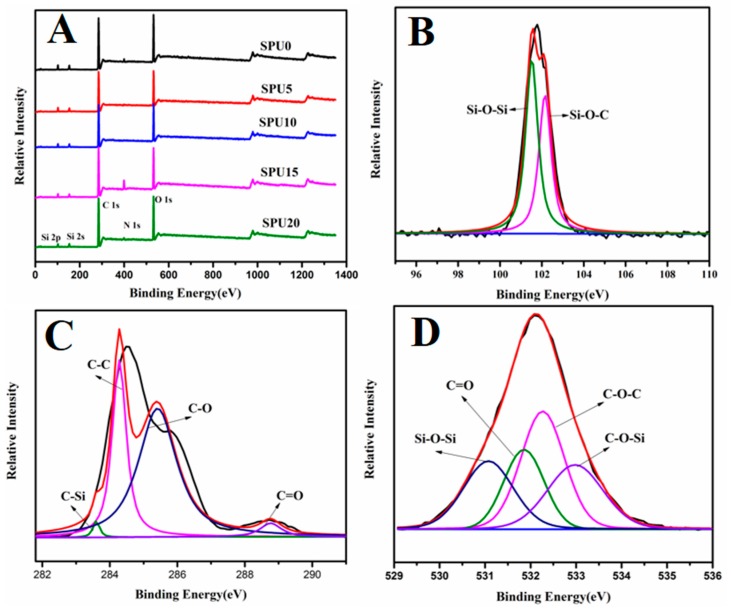
XPS spectra: (**A**) survey spectrum of different SPU films; (**B**) Si 2p of SPU15 film; (**C**) C 1s of SPU15 film; and (**D**) O 1s of SPU15 film.

The XPS survey spectra of all samples reveal the characteristic signals of carbon (C 1s), nitrogen (N 1s) and oxygen (O 1s) at about 285 eV, 400 eV and 533 eV, respectively. The existence of additional signals of silicon (Si 2p at about 102 eV and Si 2s at about 153 eV) indicates the incorporation of Si element. Figure 7 shows the XPS spectra of Si 2p on the surface of sample SPU15. The peaks at 101.95 eV and 102.6 eV are the binding energies of Si–O–Si and Si–O–C, respectively. The Si–O–C bond corresponds to triethoxysilane groups, which have survived during the film formation. The Si–O–Si bond proves the formation of the network by the condensation of the trialkoxysilane.

### 3.3. DSC Analysis of SPU Films

**Figure 8:**
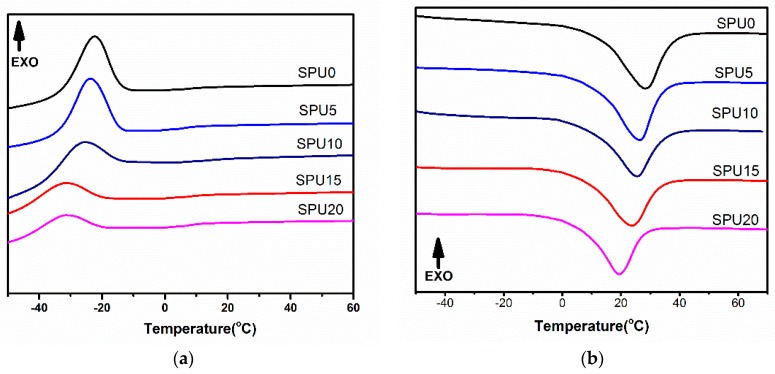
DSC thermograms of SPU films with different HDI-T contents: (**a**) curves of the cooling process; and (**b**) curves of the second heating process.

DSC results showed that the crystallization temperatures of SPU0, SPU5, SPU10, SPU15 and SPU20 were −22.4, −23.9, −25.4, −31.1, and −31.5 °C, respectively. Their melting temperatures were 29.1, 26.3, 25.7, 23.7, and 19.7 °C, respectively, as shown in Figure 8 and Table 2. The chemical linkages as a result of the polycondensation reaction between –S–O–C_2_H_5_ and H_2_O might have weakened the crystallization ability of the soft segments [36,37], decreased the melting temperature of the crystalline domains, and also restricted the movement of the molecular chains in the crystallized domains even at temperatures above the melting temperature, and provided the SPU films with an elastomer state at ambient temperature. This result indicated that the addition of HDI-T affected the crystal structure of the polymer and resulted in a lower crystallinity or lower degree of segment order.

### 3.4. X-ray Diffraction Analysis

**Figure 9:**
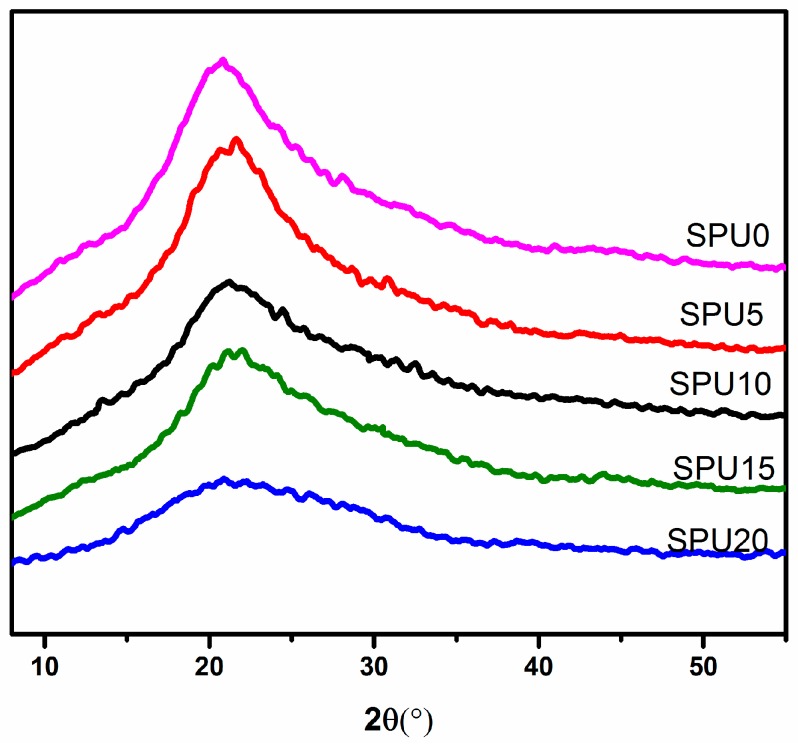
X-ray diffractograms of SPU films containing different HDI-T contents.

X-ray patterns of the SPU with different HDI-T contents are shown in Figure 9. All the diffractograms were similar and exhibited a broad diffraction halo around 22°. Moreover, the diffraction peak became weaker and broader with increasing HDI-T content, implying that the crystallinity of SPU films gradually decreased with increasing HDI-T content. The hydrolysis and condensation reaction of alkoxy silane in HDI-T and NBAPTS formed a Si–O–Si cross-linked network structure, which restricted the movement and ordered arrangement of chain segments, decreased the regularity of soft-segments, and therefore led to a decrease in the crystallinity of soft-segments [23,24,34].

### 3.5. SEM Analysis of Surface Morphologies

**Figure 10:**
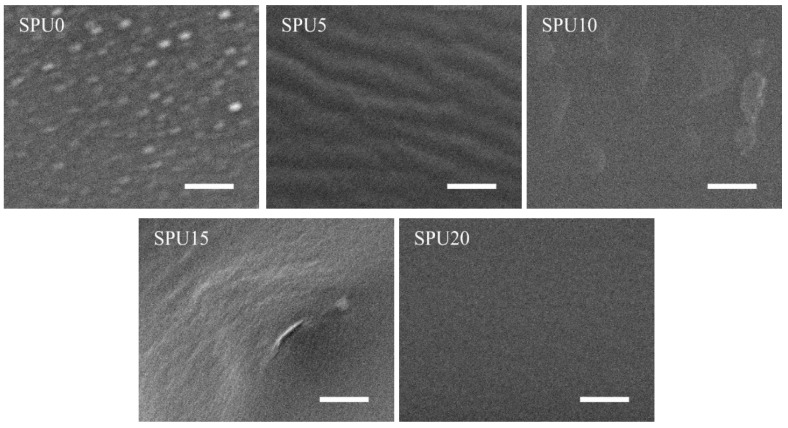
SEM micrographs of the surfaces of SPU films (scale bar = 10 μm).

Figure 10 shows the morphologies of the surfaces of the SPU films by SEM. The surface of the SPU0 was rough and contained white spots which might be siloxane particles. As the HDI-T content increased, the white spots vanished and the surface of the film became smoother. The surface of the SPU20 film was smooth and was significantly different from the films containing low HDI-T contents. It is known that microstructures of PU block copolymers can be affected by the chemical compositions and lengths of the blocks, and the miscibility between hard and soft segments [38,39]. In this work, the hard segments were composed of urethane and urea groups, and the soft ones polyester carbonyls and Si–O–Si chains. Moreover, the soft segments were the matrix and the hard ones were dispersed in it. The surfaces of the films of such materials could range from strongly phase separated to nearly homogeneous, depending on the miscibility between their soft and hard segments.

**Figure 11:**
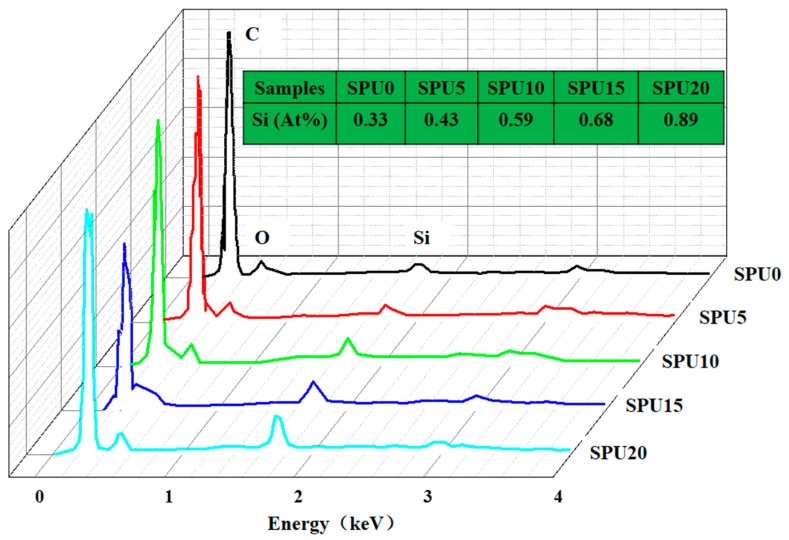
EDS analysis of different SPU films surfaces.

The EDS analysis was performed to identify the presence of atoms in the samples at a depth of 100–1000 nm from the surfaces. The expected elements (C, O and Si) can be observed in Figure 11 and Table 3. The percentage of Si element on the surface increased from 0.33% (SPU0) to 0.89% (SPU20) with increasing HDI-T content and consequently crosslinking degree. The theoretical and experimental results of EDS for the content of Si matched fairly well.

### 3.6. Thermal Properties of SPU Films

**Figure 12:**
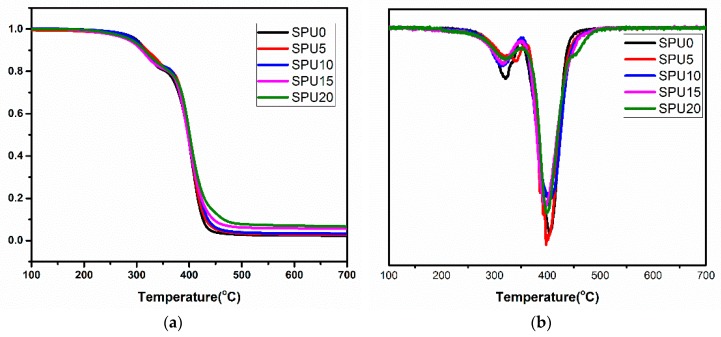
TGA (**a**) and DTG (**b**) curves of the SPU film samples.

**Figure 13:**
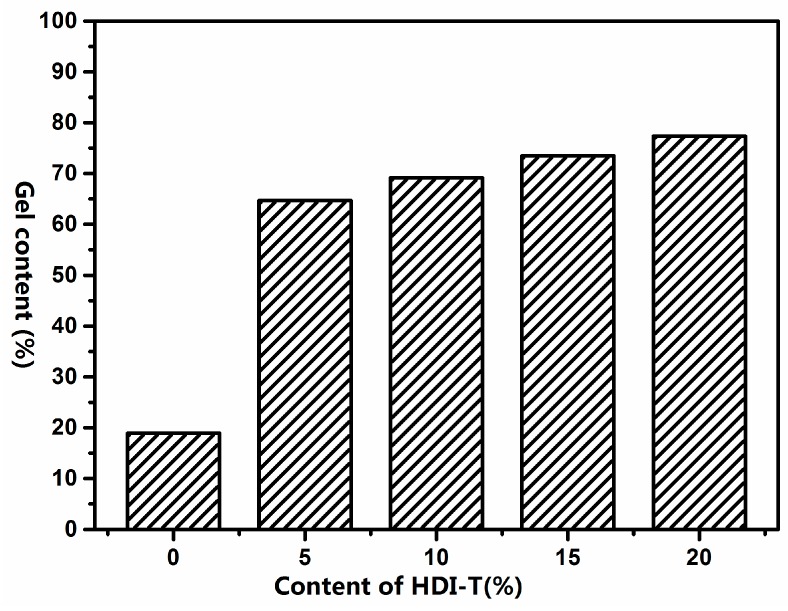
Gel contents of SPU films.

Figure 12 shows the TGA and DTG curves of all SPU hybrid films. They all showed two-stage decomposition temperatures. The slight weight loss up to 250 °C was due to the evaporation of residual moisture and the presence of organic solvents in the films [40]. The weight loss between 250 and 350 °C was attributed to the dissociation of urethane bonds to form isocyanates, alcohol and amines [41]. The degradation above 400 °C was mainly due to the scission of the cross-linked structure. The major decomposition product was SiO_2_. The DTG curves of the SPUs shifted slightly to a lower temperature with an increase in HDI-T content, which was slightly different from some of the research works reported in the literature [39]. Two factors might be responsible for this phenomenon. On the one hand, as HDI-T content increased, the gel content of SPU increased as shown in Figure 13. That was beneficial for the thermal stability. On the other hand, the content of C–N bond increased with increasing HDI-T content. However, the bond energy of C–N (305 kJ·mol^−1^) was lower than those of C–C (346.9 kJ·mol^−1^) and C–H (414 kJ·mol^−1^). That was unfavorable for the thermal stability. Therefore, the thermal stability of the SPU films was a trade-off between these two opposite factors.

### 3.7. Mechanical Properties of SPU Films

**Figure 14:**
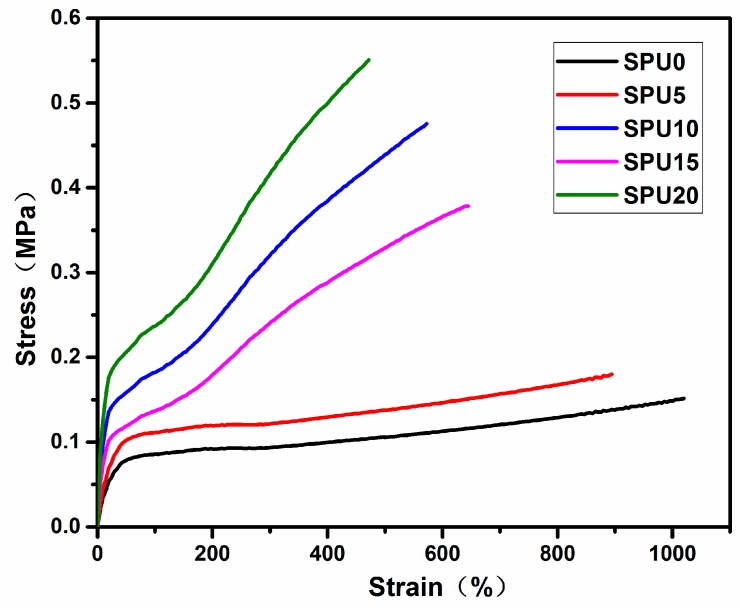
Tensile stress–strain curves of SPU with different HDI-T contents.

Figure 14 shows that the tensile strength of the SPU films increased with increasing HDI-T content, whereas their elongation at break showed an opposite trend because of an increase in the crosslinking degree of SPU films with the formation of Si–O–Si linkage through the hydrolysis and condensation process. Table 4 summarizes the Young’s modulus, tensile strength, elongation at break and pencil hardness values as a function of HDI-T content. When HDI-T content was increased from 0 to 20 mol %, the Young’s modulus and tensile strength were increased from 0.41 MPa to 1.77 MPa and from 0.15 MPa to 0.55 MPa, respectively. Meanwhile, the elongation at break decreased from 1019.7% to 471.4%, suggesting an increased brittleness. All those changes resulted from the increased crosslinking degree. The crosslinking reduced the mobility of the chains during tensile deformation and consequently increased their mechanical properties [42,43]. The hardness increased from HB to 2H with increasing HDI-T content.

### 3.8. Surface Property and Water Absorption of SPU Films

**Figure 15:**
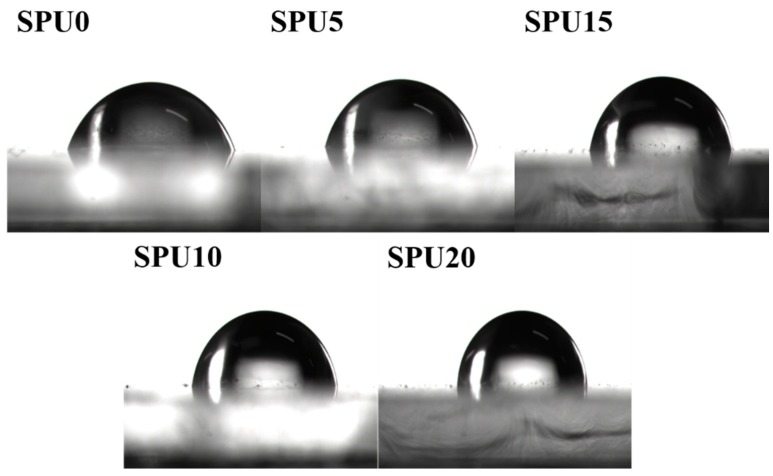
Contacts angle of water on SPU films.

The contact angle of water test was used to characterize the surface properties of SPU films. The results are shown in Figure 15. As the HDI-T content increased, the contact angle of water on the SPU film increased. It was 66.5°, 71.5°, 79.2°, 81.2° and 86.5° for SPU0, SPU5, SPU10, SPU15 and SPU20, respectively, indicating an obvious improvement in hydrophobicity of the SPU films. The results indicated that the incorporation of alkoxysilane reduced the surface free energy of the SPU films because of the migration of Si atoms with a low polarity to the surfaces of the SPU films [44]. Therefore, the wettability of the SPU films decreased and their hydrophobicity increased with increasing alkoxysilane content. This phenomenon agreed with the results reported in the literature [45,46,47].

**Figure 16:**
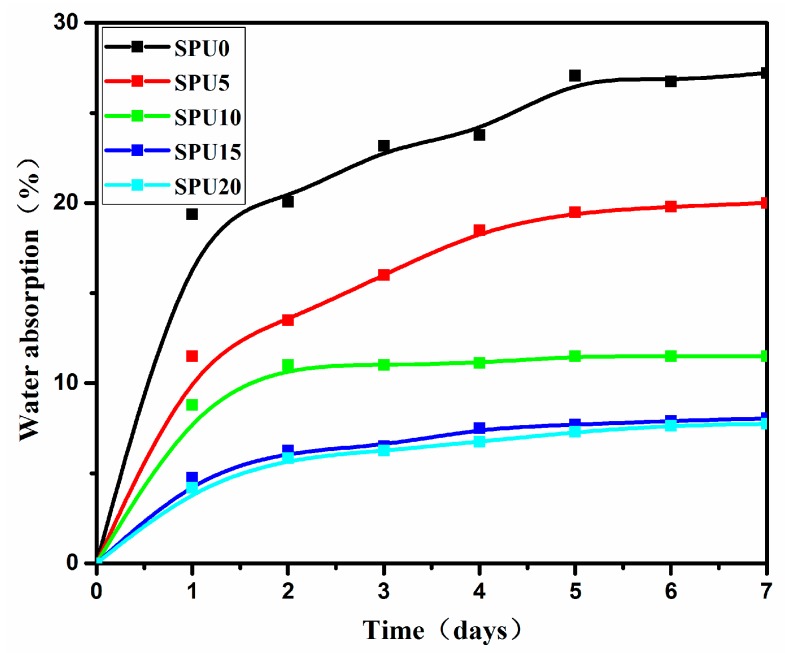
Water absorption of SPU films as a function of time in days.

In parallel with the improved surface hydrophobicity with increasing HDI-T content from 0% to 20%, the water absorption of the SPU films after seven days decreased from 27.2% to 7.6%, as shown in Figure 16. This indicates that the incorporation of HDI-T in SPU improved their water resistance. This may be ascribed to the formation of a hydrophobic layer of Si–O–Si chains enriched on the surface of SPU film and that of a crosslinked siloxane network structure, both of which prevent water molecules from getting into and diffusing through the film.

## 4. Conclusions

In this study, we synthesized HDI-T and incorporated it into SPU chains. HDI-T content was between 0 and 20 mol %. FT-IR, XPS and XRD results showed successful incorporation of HDI-T into polyurethanes and the formation of Si–O–Si. SEM images exhibited a much smoother surface and EDS test found the Si element content increased as the increase of HDI-T content, DSC demonstrated that both crystallization temperature and melting temperature moved to a lower point as a result of the incorporation of HDI-T. The resulted SPU coating films exhibited an increased surface hydrophobicity and decreased water adsorption. Meanwhile, the Young’s modulus, tensile strength and pencil hardness of the films were also improved with increasing HDI-T content, but their elongation at break decreased. The thermal stability of the SPU films became poorer with the incorporation of more C-N bond as a result of an increase in HDI-T content. This study provided a new effective way to prepare SPU coating materials with different performances.

## Figures and Tables

**Table 1:** Compositions for the synthesis of SPU hybrids.

Samples	HDI (mol)	HDI-T (mol)	PTMG (mol)	BDO (mol)	NBAPTS (mol)
SPU0	0.0480	0	0.02	0.02	0.016
SPU5	0.0456	0.0024	0.02	0.02	0.016
SPU10	0.0432	0.0048	0.02	0.02	0.016
SPU15	0.0408	0.0072	0.02	0.02	0.016
SPU20	0.0384	0.0096	0.02	0.02	0.016

**Table 2:** Crystallization temperature and melting temperature of different SPU films.

Sample	SPU0	SPU5	SPU10	SPU15	SPU20
Crystallization temperature (°C)	−22.4	−23.9	−25.4	−31.1	−31.5
Melting temperature (°C)	29.1	26.3	25.7	23.8	19.8

**Table 3:** Theoretical and experimental EDS results of SPU films.

Sample	Si Concentration/%	
	Theoretical	Experimental
SPU0	0.40	0.33
SPU5	0.45	0.43
SPU10	0.50	0.59
SPU15	0.55	0.68
SPU20	0.60	0.89

**Table 4:** Mechanical properties of SPU films with different HDI-T contents.

HDI-T Content (%)	Young’s Modulus (MPa)	Tensile Strength (MPa)	Elongation at Break (%)	Pencil Hardness
0	0.41	0.15	1019.7	HB
5	0.53	0.18	894.6	H
10	1.02	0.38	644.5	2H
15	1.36	0.48	572.3	2H
20	1.77	0.55	471.4	2H
